# Effects of the COVID-19 pandemic on surgical treatment for thoracic malignant tumor cases in Japan: a national clinical database analysis

**DOI:** 10.1007/s00595-024-02907-w

**Published:** 2024-12-07

**Authors:** Yasushi Shintani, Hiroyuki Yamamoto, Yukio Sato, Masayoshi Inoue, Keisuke Asakura, Hiroyuki Ito, Hidetaka Uramoto, Yoshinori Okada, Toshihiko Sato, Mariko Fukui, Yasushi Hoshikawa, Toyofumi Fengshi Chen-Yoshikawa, Masayuki Chida, Norihiko Ikeda, Ichiro Yoshino

**Affiliations:** 1 Department of General Thoracic Surgery, Osaka University Graduate School of Medicine, 2-2-L5, Yamadaoka, Suita, Osaka 565-0871 Japan; 2 Department of Healthcare Quality Assessment, The University of Tokyo, Tokyo, Japan; 3 Department of Thoracic Surgery, University of Tsukuba, Ibaraki, Japan; 4 Division of Thoracic Surgery, Department Surgery, Kyoto Prefectural University of Medicine, Kyoto, Japan; 5 Division of Thoracic Surgery, Department of Surgery, Keio University School of Medicine, Tokyo, Japan; 6 Department of Thoracic Surgery, Kanagawa Cancer Center, Kanagawa, Japan; 7 Department of Thoracic Surgery, Kanazawa Medical University, Ishikawa, Japan; 8 Department of Thoracic Surgery, Tohoku University, Miyagi, Japan; 9 Department of General Thoracic, Breast and Pediatric Surgery, Fukuoka University School of Medicine, Fukuoka, Japan; 10 Department of General Thoracic Surgery, Juntendo University, Tokyo, Japan; 11 Department of Thoracic Surgery, Fujita Health University School of Medicine, Aichi, Japan; 12 Department of Thoracic Surgery, Nagoya University Graduate School of Medicine, Aichi, Japan; 13 Department of General Thoracic Surgery, Dokkyo Medical University, Tochigi, Japan; 14 Department of Surgery, Tokyo Medical University, Tokyo, Japan; 15 Department of General Thoracic Surgery, Chiba University Graduate School of Medicine, Chiba, Japan; 16 Department of Thoracic Surgery, International University of Health and Welfare School of Medicine, Chiba, Japan

**Keywords:** COVID-19, Lung cancer, Mediastinal tumor

## Abstract

**Objective:**

Surgical care has been significantly affected by the COVID-19 pandemic. This study was conducted to evaluate the effects of the pandemic on lung cancer and mediastinal tumor surgery.

**Methods:**

Changes in the number of surgical procedures for lung cancer and mediastinal tumors were analyzed using the National Clinical Database of Japan. Patient characteristics, including disease stage and histological type, from 2019 to 2022 were evaluated using annual datasets.

**Results:**

Comparisons with 2019 showed that the number of patients who underwent surgery for primary lung cancer or a mediastinal tumor decreased in 2020 and then remained stable. There were no clinically significant changes in the trend over the four-year period regarding the number of patients for each clinical and pathological stage of lung cancer. Regarding mediastinal tumors, there was no significant difference in tumor size between years. There was a slight change in the selection of surgical indication during the second quarter of 2020, although its impact on annual trends in the stage distribution for lung cancer and primary disease for mediastinal tumors was minimal.

**Conclusions:**

Analyses of lung cancer and mediastinal tumor surgery cases in Japan during the COVID-19 pandemic showed no significant disease profile changes related to treatment delay.

**Supplementary Information:**

The online version contains supplementary material available at 10.1007/s00595-024-02907-w.

## Introduction

The novel coronavirus responsible for the COVID-19 pandemic, SARS-CoV-2, was first identified in Wuhan, China in December 2019 and then rapidly spread around the world [1]. Health resources have channeled their services toward COVID-19 patient care, resulting in many non-COVID-19-related care activities, including disease diagnosis and therapeutic services, becoming postponed or canceled, with a tremendous impact on patient services [2]. At the time of writing, 10 waves of COVID-19 infection have affected Japan. The national government declared an initial state of emergency in response to the first wave in April 2020, which resulted in a significant reduction in the number of surgical operations [3]. In all countries, cancer surgery systems became fragile during lockdowns, with delays and canceled operations for cancer treatment, which may have led to the long-term reductions in survival [4]. Reports of clinical experience by Japanese thoracic surgeons also indicate a decrease in the number of general thoracic surgical procedures, including those for lung cancer, in 2020 and 2021 [5, 6].

Participation in the Japanese cancer screening program has also seen a decline since the start of the COVID-19 pandemic [7], and resultant delays could result in a significantly increased number of cases of more advanced disease. The greater number of patients receiving late-stage diagnoses during increases in pandemic-related cases may reflect findings showing that only sick patients with symptoms and/or acute events that required immediate care sought hospital attention, resulting in shifts in disease stage at the time of the diagnosis associated with the pandemic [8]. In addition, there was a significant disruption in lung cancer screening, leading to a decrease in new patients being screened and a consequent increased proportion of nodules found that were possibly malignant once screening resumed [9]. Postponing diagnosis and resection can lead to upstaging and a reduced survival, even in patients with early-stage non-small-cell lung cancer (NSCLC) [10]. Furthermore, barriers blocking the lung cancer screening care continuum, including delays in follow-up appointments, can reduce effectiveness [11], which is likely associated with the clinical upstaging of patients with positive findings of lung cancer as well as other thoracic malignancies, such as malignant mediastinal tumors.

Several studies have shown a decreased number of cases of newly detected lung cancer during the COVID-19 pandemic, especially in the early period [12]. In December 2020, COVID-19 vaccines were introduced in Japan and demonstrated greater than 90% efficacy in preventing infections [13]. The prevalent availability of vaccines and other effective therapies for COVID-19 might relieve the hesitancy shown by individuals to seek necessary healthcare services as well as treatment.

To clarify the status of thoracic surgery, the present study was performed using the National Clinical Database of Japan (NCD), a nationwide web-based surgical patient registry system, to evaluate the impact of the pandemic on the number of surgical procedures for lung and mediastinal tumors. In addition, trends related to the stage distribution for lung cancer and primary disease for mediastinal tumor cases during the long-term COVID-19 pandemic in Japan were examined.

## Patients and methods

To investigate the impact of the COVID-19 pandemic on surgical treatments for thoracic malignant tumors, a number of primary lung cancer and mediastinal tumor surgery cases in 2022 were compared with those from 2014 to 2021 using the NCD. The data registration system and recorded information are described in detail in our previous report [14]. The NCD system is used for board certification as the second level of hierarchy for the specialty of chest surgery and accreditation by associated educational institutions and is considered to be well organized, with high rates of registration and input accuracy.

Fundamental and perioperative factors for each disease were also analyzed using annual datasets for four years from 2019 to 2022, which included information regarding cases of surgery for primary lung cancer or mediastinal tumor. Patient and tumor characteristics, including sex, age, surgical procedure, surgical approach, stage, and histological type, as well as quarterly trends in these factors were evaluated.

Statistical analyses were performed as previously reported [5, 6]. Numeric data are expressed as median values (interquartile range) for non-normal distributions, whereas categorical data are expressed as percentages. This study was approved by the institutional review board of Osaka University Hospital (#23,178).

## Results

### Annual changes from 2014 to 2022

**Fig. 1:**
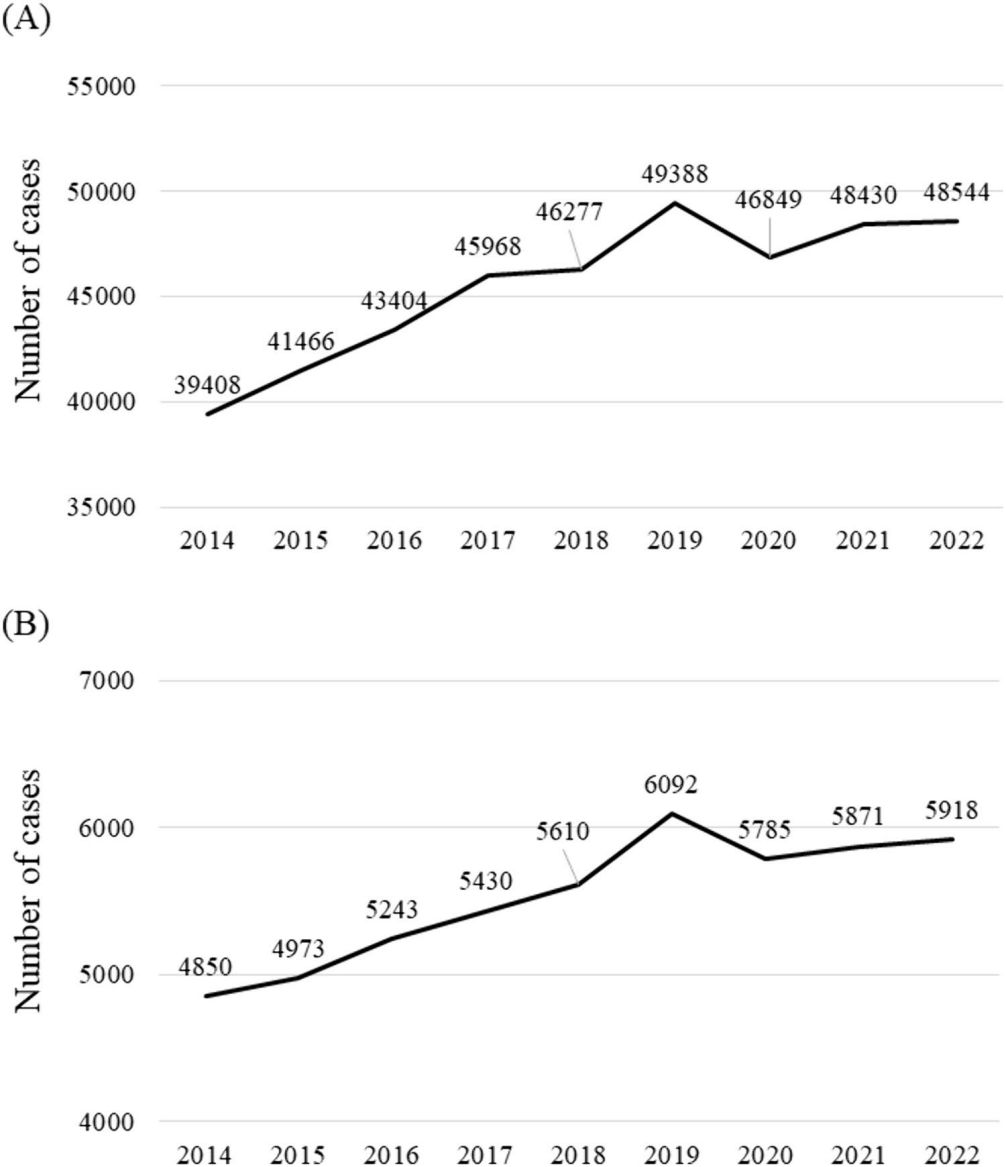
Trends in numbers of surgical procedures for **A** primary lung cancer and **B** mediastinal tumor cases from 2014 to 2022

The number of patients who underwent surgery for primary lung cancer decreased in 2020 and remained stable thereafter (Fig. 1A). More precisely, the number of patients who underwent surgery for primary lung cancer in 2022 was 1.7% lower than that in 2019 (from 49,388 to 48,544). Sato et al. reported an annual increase of 5.0% in the number of surgeries for lung cancer from 2014 to 2019 [6], in contrast to a considerable decrease of more than 15% (Fig. 1A). Similarly, the number of surgical procedures for mediastinal tumors in 2022 decreased by 2.8% compared to 2019 (from 6092 to 5918), in contrast to the 5.0% annual increase from 2014 to 2019, thus again showing a considerable decrease of 15% (Fig. 1B).

### Clinicopathological features of lung cancer patients

The patient characteristics are shown in Table S1. Background factors, including age, sex, and performance status, did not differ markedly among the analyzed years.

**Fig. 2:**
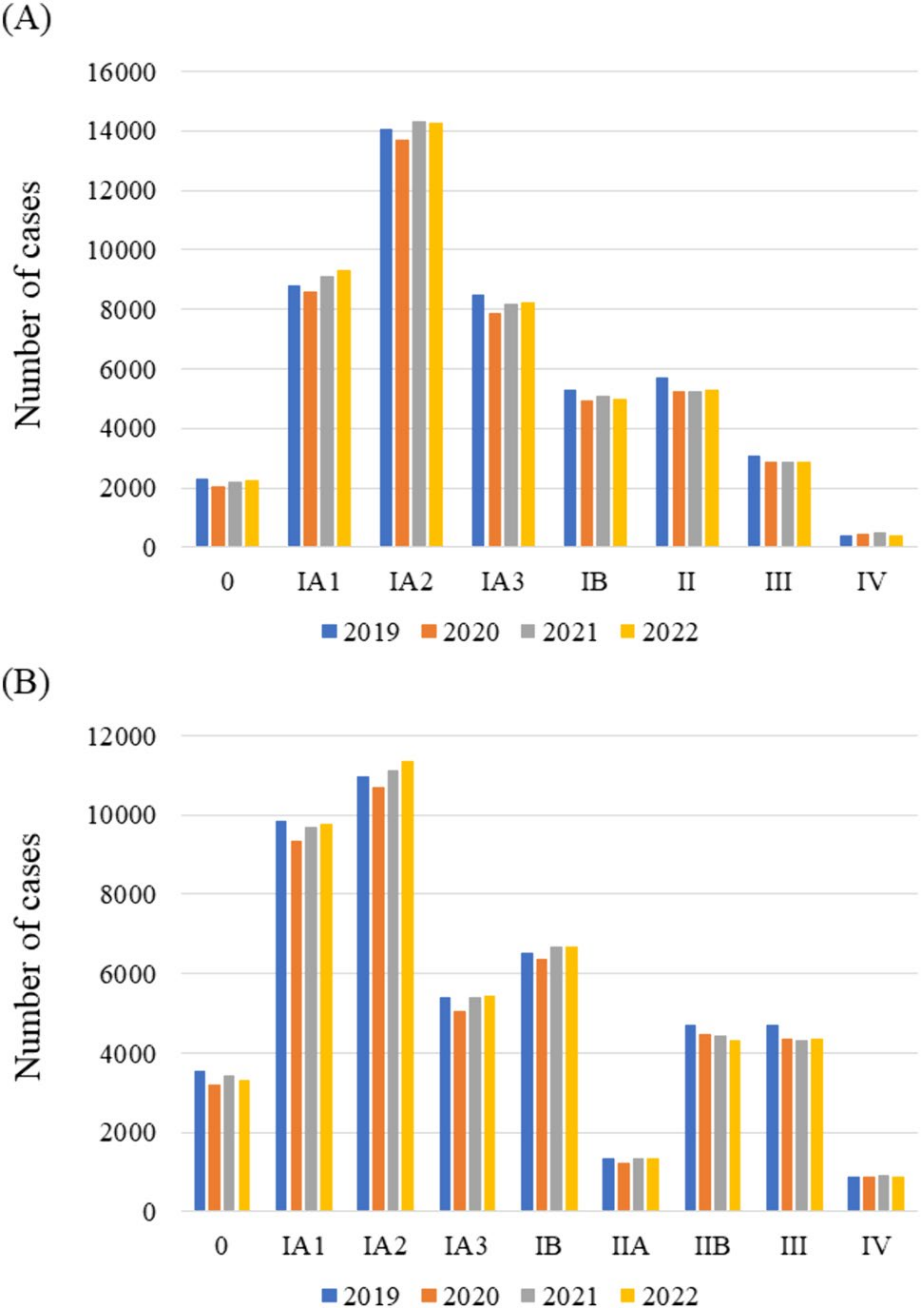
Trends in lung cancer **A** clinical and **B** pathological stages from 2019 to 2022

Trends in the number of patients who underwent surgery from 2019 to 2022 for each clinical and pathological stage of lung cancer over time are shown in Fig. 2. As compared to the number of cases in 2019, that was decreased in 2020 for all clinical stages except stage IV, while the numbers of IA1 and IA2 patients were increased in 2021 and 2022 (Fig. 2A, Table S1). When tumor and invasion sizes were compared among years, there was no significant change; thus, the proportions of patients with each clinical T status were nearly the same. The proportion of patients who underwent robot-assisted thoracic surgery (RATS) increased each year, while that of video-assisted thoracic surgery (VATS) cases decreased, and that of open surgery cases increased (Table S2). Furthermore, the proportion of patients who underwent segmentectomy increased each year, while that of lobectomy cases decreased. There were no remarkable changes in the rates of postoperative pulmonary complications or 30-day mortality among the examined years (Table S2). In addition, there was no significant change in the histologic type of lung cancer, tumor, or invasion size from 2019 to 2022 (Table S3). Pathological IA2, IA3, and IB cases showed increases in 2021 and 2022 compared to 2019, while stage II and III cases decreased from 2020 to 2022 compared to 2019 (Fig. 2B, Table S3). No annual clinical or pathological stage shift was noted in the patients who underwent surgery for lung cancer between 2019 and 2022.

**Fig. 3:**
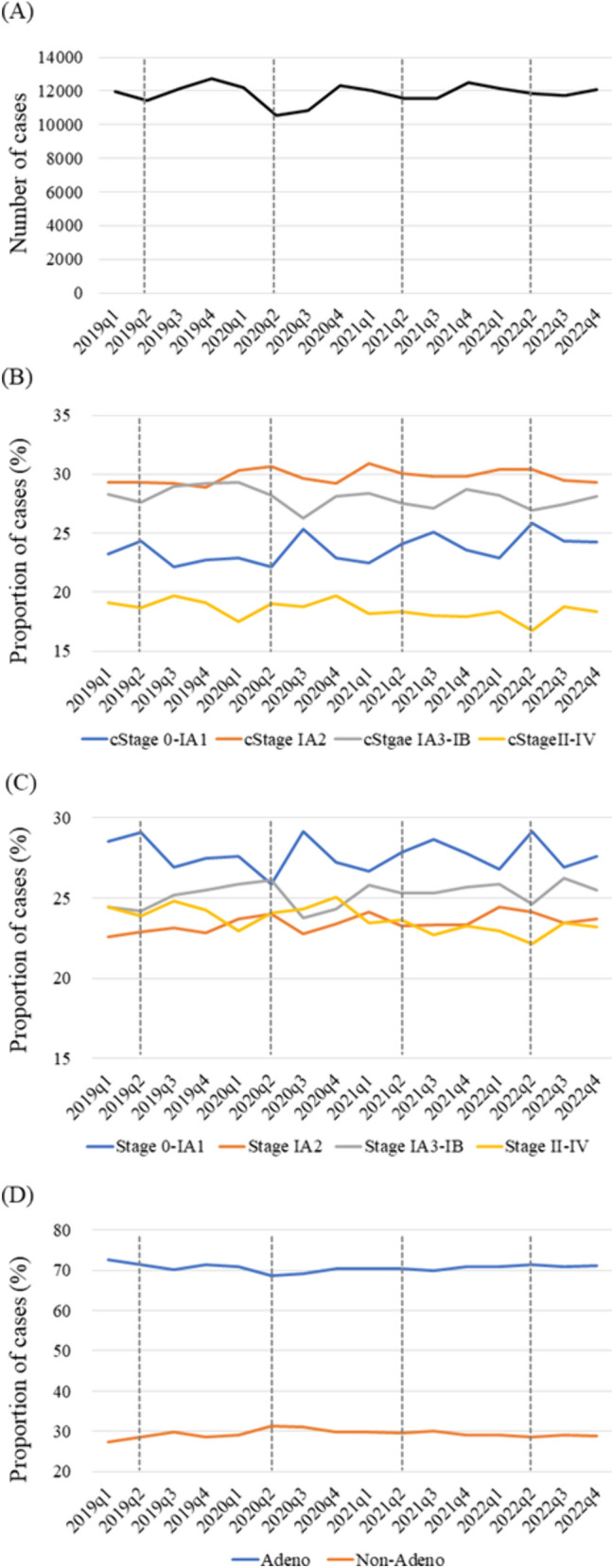
Trends in **A** numbers of patients who underwent surgery for lung cancer as well as the **B** clinical stage, **C** pathological stage, and **D** histological type during each quarter (q) from 2019 to 2022. Adeno: adenocarcinoma, Non-adeno: non-adenocarcinoma

The number of cases of lung cancer surgery on a quarter-year basis is shown in Fig. 3A. The number of surgical cases in quarter 2 (Q2) of 2020 was remarkably lower than that in the other investigated periods. While the proportions of clinical and pathological stage 0–IA1 cases decreased in Q2, they increased in Q3 (Fig. 3B,C). In contrast, the proportion of non-adenocarcinoma cases increased in Q2 in 2020 (Fig. 3D).

### Clinicopathological features of mediastinal tumor patients

**Fig. 4:**
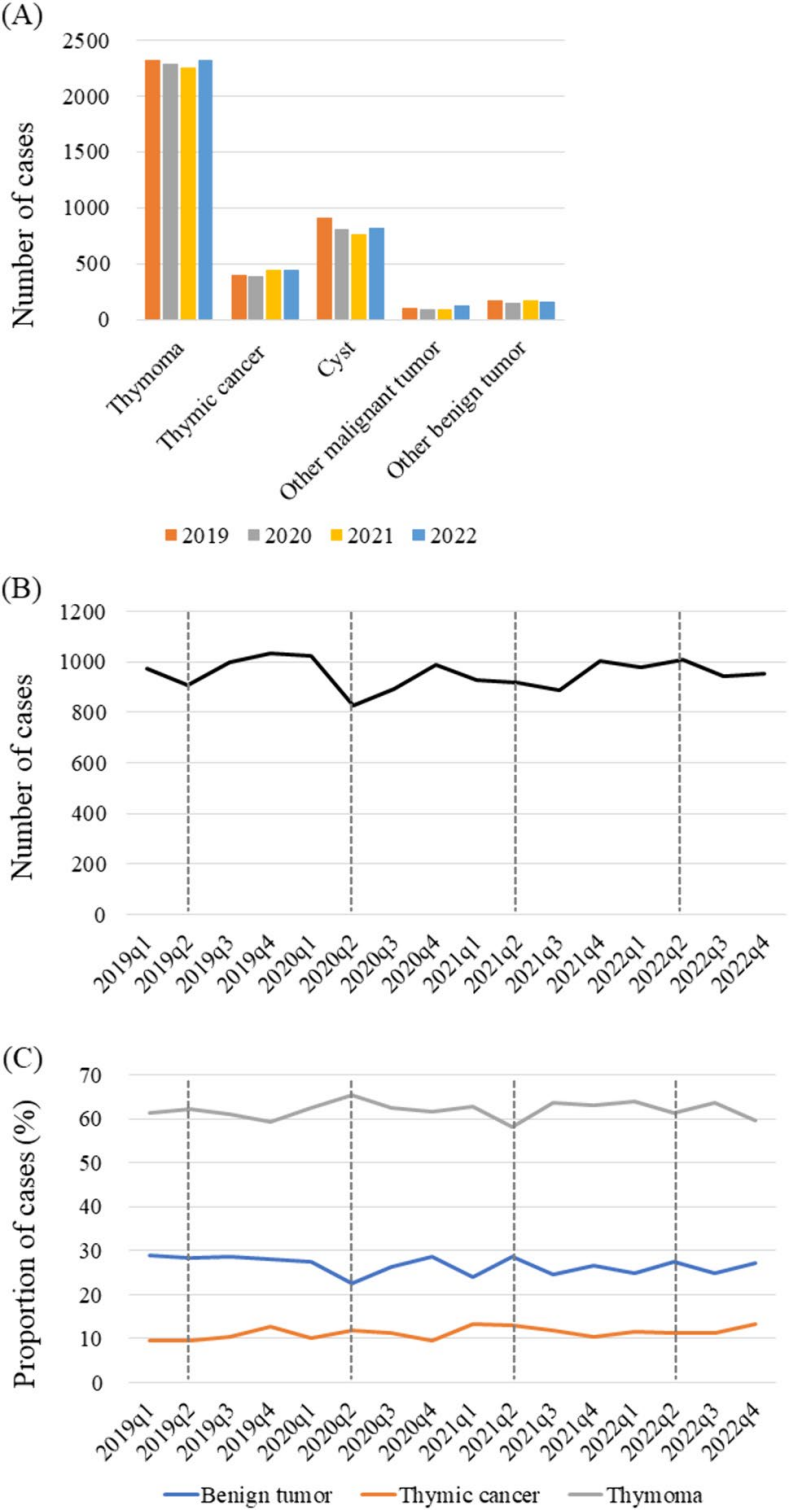
Trends in numbers of patients **A** with mediastinal tumors according to histological findings, as well as **B** those who underwent surgery for mediastinal tumors and **C** histological type in each quarter (q) from 2019 to 2022

The patient characteristics are shown in Table S4. Background factors, including age, sex, and performance status, were not significantly different between the examined years (Table S4). Similar to lung cancer surgery, the proportion of patients who underwent RATS increased each year, whereas the proportion of VATS and open surgery cases decreased. No marked changes in the rate of postoperative pulmonary complications, 30-day mortality, or tumor size were noted among the examined years (Table S5). While the number of cases of surgery for thymic malignancy, such as thymoma or thymic carcinoma, remained stable, that for cystic disease decreased from 2020 to 2022 compared to 2019 (Fig. 4A, Table S5).

Finally, the number of surgical cases in Q2 of 2020 was markedly lower than that in the other examined periods (Fig. 4B). In contrast, there was an increased proportion of thymic malignancy and a decreased proportion of benign mediastinal tumor cases in Q2 of 2020 (Fig. 4C).

## Discussion

Several aspects related to the management of patients with cancer have been affected by the COVID-19 outbreak. The present study was conducted to examine the volume of surgical procedures and perioperative factors, including stage and histology, for lung cancer and mediastinal tumor cases during the long-term COVID-19 pandemic in Japan. According to the NCD, the number of surgeries for lung cancer and mediastinal tumors decreased in 2020 and did not recover until 2022. A possible main reason is the decrease in the number of diagnostic procedures conducted for thoracic malignancy due to fewer patients undergoing cancer screening and postponed diagnostic evaluations during the early pandemic period [5, 15]. It is also conceivable that a large number of individuals avoided undergoing regular medical checkups because of the pandemic situation, even in 2021 and 2022. There were widespread concerns regarding the increased risk of infection when visiting a medical facility or hospital, as well as regarding the increased burden on healthcare workers, even after the introduction of vaccines and other effective therapies for treating COVID-19.

**Fig. 5:**
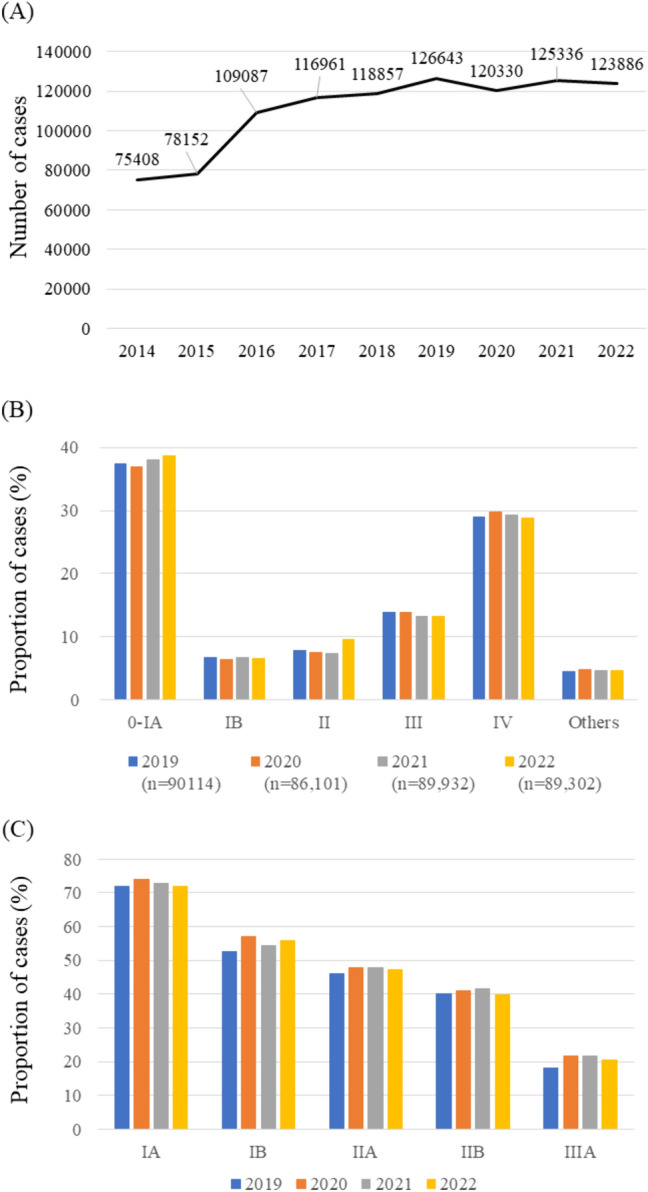
**A** Trend in numbers of patients with primary lung cancer from 2014 to 2022 calculated from data published in the Annual Report of Hospital-Based Cancer Registries. **B** Trends in the clinical stage of lung cancer from 2019 to 2022. **C** Trends in the rate of surgical cases according to clinical stage of lung cancer from 2019 to 2022

The hospital-based cancer registry (HBCR) data presented by the National Cancer Center for Japan showed that the number of lung cancer patients steadily increased from 2014 to 2019 and decreased in 2020, remaining broadly flat (Fig. 5A). HBCRs are used in cancer care hospitals designated by the Ministry of Health, Labour, and Welfare as a condition of designation, and the data therein are estimated to represent approximately 80% of incident cases in Japan, including surgical and non-surgical patients [16]. While the exact numbers for all such Japanese cases may not be fully presented, there was no marked increase in the data. Currently, there is an ongoing increase in the number of older individuals in Japan (≥ 65 years old) that is forecasted to peak by 2042 [17]. Thus, since the incidence rate of lung cancer is age dependent, it is expected that the number of lung cancer cases will also increase. It is therefore possible that the number of patients with undiagnosed lung cancer will sharply increase in the near future.

Regarding mediastinal tumors, precise numbers related to the incidence of each mediastinal disease during the COVID-19 pandemic have not been presented because of rarity, and the present report is the first to describe the number of surgeries for mediastinal tumors, including thymic malignancy. It is not uncommon for a mediastinal tumor mass to be asymptomatic in the long term, with diagnoses following incidental findings frequently noted [18], underscoring the importance of medical checkups for early detection. Similar to lung cancer, the estimated rate of decrease in the number of surgical procedures for mediastinal tumors during the pandemic was 15%; thus, it is possible that, in the near future, the number of patients with previously undiagnosed mediastinal tumors will also increase.

Reyes et al. reported that 38% fewer new lung cancer cases were diagnosed compared to prior to the COVID-19 pandemic period, while there were more cases of symptomatic and severe lung cancer [19]. Furthermore, undiagnosed thoracic malignant diseases are anticipated to be revealed at a more advanced stage, resulting in an increased number of cases with a worse prognosis. These factors are associated with a significant risk of delays in the diagnosis and/or access to treatment, which may result in suboptimal therapeutic care for cancer patients, consequently contributing to increased mortality. Thus, the present study was conducted to evaluate the trends in the stage distribution of lung cancer and primary diseases related to mediastinal tumors.

There was no significant change in the clinical or pathological stage of the surgical cases of lung cancer during the period examined in this study. Notably, there have been no reports regarding the number of surgeries for thoracic malignancies during the long-term COVID-19 pandemic, and the present study is the first to show the current situation in Japan. According to data from HBCRs, no annual shift in the clinical stage of lung cancer, including surgical and non-surgical cases, has occurred (Fig. 5B). Furthermore, the annual rate of surgical cases at each stage was nearly the same from 2019 to 2022, indicating that the choice of treatment did not change during the pandemic (Fig. 5C). Japan’s statutory health insurance system provides universal coverage for all residents. During the pandemic, healthcare services were well maintained for patients with lung cancer and those affected by coronavirus infection. In addition, because computed tomography (CT) screening, which has an excellent ability to detect early lung cancer, is performed more frequently in Japan than in other countries, there are a significant number of incidental lung cancer diagnoses [20]. Consequently, the impact of the coronavirus pandemic on medical conditions varied among countries, not only for patients receiving care but also among healthcare systems and other related factors. However, although the number of surgeries for thymoma did not change, those for thymic cancer increased throughout the study period, while those for cysts, which are apparently benign disease cases, decreased (Fig. 4A). These findings are likely due to the selection of surgical indications according to the preoperative diagnosis of mediastinal disease.

The number of surgical cases of lung cancer and mediastinal tumor in Q2 of 2020 was markedly lower than that during any other period, which is in correlation with the first declaration of the state of emergency in Japan, from April 7 to May 25 of that year. While the rate of pathological stage 0–IA1 lung cancer cases decreased in Q2 and then increased in Q3 of 2020, that of stage IA2–IB cases decreased in Q3 (Fig. 3C). Similarly, the rate of adenocarcinoma cases decreased while that of non-adenocarcinoma cases increased in Q2 (Fig. 3D), likely because non-adenocarcinoma cases are determined based on the detection of an invasive tumor, which is more frequently noted in chest X-ray findings than in adenocarcinoma. Small peripheral lung adenocarcinomas with ground-glass nodules have been reported to be difficult or even impossible to detect using routine chest radiography [21]. Due to the pandemic, many diagnostic procedures, including chest CT and biopsy examinations, have been delayed [22]. Another possibility is that surgery for patients with early-stage adenocarcinomas was often postponed because of the first declaration of the state of emergency. Kato et al. reported that pathological outcomes of patients with stage I lung cancer during the early pandemic period tended to include larger tumors and invasive size due to surgery only being indicated for lung cancer with high malignancy [23]. Furthermore, Mayne et al. evaluated the impact of an extended delay to conduct surgery for stage I NSCLC and concluded that a delayed procedure was associated with a worse prognosis for stage IA2–IB adenocarcinoma and stage IB squamous cell carcinoma but not for stage IA1 adenocarcinoma or stage I squamous cell carcinoma [24]. The present findings also indicate that patients with early-stage lung cancer underwent surgery immediately after the state of emergency was declared, suggesting that delayed surgery for stage 0–IA1 lung cancer does not have an effect on the prognosis. The rate of surgical procedures for benign mediastinal tumor cases also decreased in Q2 of 2020, probably for the same reason as noted for lung cancer surgery.

There were no marked changes in the rates of postoperative pulmonary complications or 30-day mortality during the COVID-19 pandemic. Another study that used NCD data found that the mortality and morbidity rates in patients undergoing distal gastrectomy for gastric cancer were not worse during the pandemic than during the pre-pandemic period [25]. These results suggest that appropriate perioperative management is required for patients with cancer in Japan.

The decrease in VATS and increase in RATS procedures for lung cancer and mediastinal tumor cases might have reflected the spread of RATS following its approval as an insurable procedure by the National Health Insurance System in April 2018. Similarly, the increase in segmentectomy procedures for lung cancer may have been due to changes in surgical method selection based on the results of the Japan Clinical Oncology Group (JCOG) series (JCOG0802/WJOG4607L and JCOG1211), which showed that sublobar resection for small peripheral NSCLC could be considered an oncologically effective alternative to a lobectomy procedure [26, 27]. While the reasons for the increase in the number of open surgery procedures for lung cancer are unclear, reasonable speculation is possible, as patients with advanced lung cancer did not increase during the period. First, guidelines released by surgical societies early in the pandemic recommended open surgery over endoscopic surgery as a way of reducing the transmission of COVID-19 via surgical smoke [28]. Another possibility is that open surgery was favorably selected in consideration of surgical safety, and limits on the number of healthcare workers available during the pandemic may also have played a part.

Several limitations associated with the present study warrant mention. First, due to the retrospective nature and use of data obtained from the NCD, selection bias could not be avoided, and only short-term surgical data were evaluated. Although no increase in cases of advanced thoracic malignancy was found to be associated with the COVID-19 pandemic, the analyzed population included patients with a short-term follow-up period. Second, the study period was limited to 2019–2022. The legal classification of COVID-19 was downgraded to “Class 5” in Japan on May 8, 2023, placing it in the same category as common infectious diseases, such as seasonal influenza. Nevertheless, waves of increased COVID-19 infections were noted in 2022 and 2023, so it cannot be denied that the effects of the pandemic on surgery for thoracic malignant tumor cases will continue in the future. Additional studies are necessary to clarify the future effects of COVID-19 on trends in the stage distribution for lung cancer and primary disease in mediastinal tumor cases in Japan.

In conclusion, there were no marked changes in disease progression in thoracic malignancy cases that caused treatment delays during the prolonged COVID-19 pandemic in Japan. However, since the number of surgical procedures for lung cancer and mediastinal tumors decreased in 2020 and did not fully recover by 2022, there is a possibility that an increase in patients with these conditions may occur in the near future. Further research is required to better understand the long-term impact of the pandemic on the burden of care for related diseases.

## Supplementary Information

Below is the link to the electronic supplementary material.

Supplementary file1 (DOCX 44 KB)

## Data Availability

Based on the data use policy of the JACS, data access can be approved following an assessment by the JACS NCD committee. Those interested in using the data should contact the JACS NCD Committee (jacs-soc@umin.ac.jp) to submit their proposals. The data will be granted after approval.
