# Supplementary material for: Effects of the COVID-19 pandemic on surgical treatment for thoracic malignant tumor cases in Japan: a national clinical database analysis
Source: Surg Today. 2024 Dec 7;55(2):265–72. doi: 10.1007/s00595-024-02907-w (PMC11757856; doi:10.1007/s00595-024-02907-w)
Supplement: Supplementary file 1 — Supplementary file1 (DOCX 44 KB) [file 595_2024_2907_MOESM1_ESM.docx]

Table S1. Characteristics of lung cancer patients

|  |  | 2019 | | 2020 | | 2021 | | 2022 | |
| --- | --- | --- | --- | --- | --- | --- | --- | --- | --- |
| Age | -59 | 5,627 | (11.6%) | 5,058 | (11.0%) | 5,486 | (11.5%) | 5,359 | (11.2%) |
|  | 60-69 | 12,892 | (26.7%) | 11,597 | (25.3%) | 11,271 | (23.6%) | 10,865 | (22.7%) |
|  | 70-79 | 22,997 | (47.6%) | 22,616 | (49.3%) | 23,781 | (49.9%) | 23,943 | (50.1%) |
|  | 80- | 6,801 | (14.1%) | 6,647 | (14.5%) | 7,122 | (14.9%) | 7,641 | (16.0%) |
| Gender | Female | 19,150 | (39.6%) | 17,786 | (38.7%) | 18,680 | (39.2%) | 19,358 | (40.5%) |
|  | Male | 29,167 | (60.4%) | 28,132 | (61.3%) | 28,980 | (60.8%) | 28,450 | (59.5%) |
| Performance status | PS0 | 40,591 | (84.0%) | 38,558 | (84.0%) | 40,273 | (84.5%) | 40,959 | (85.7%) |
|  | PS1 | 5,951 | (12.3%) | 5,630 | (12.3%) | 5,464 | (11.5%) | 5,142 | (10.8%) |
|  | PS2-4 | 1,775 | ( 3.7%) | 1,730 | ( 3.8%) | 1,923 | ( 4.0%) | 1,707 | ( 3.6%) |
| Tumor size (cm) | Total size | 2.2 | (1.5-3.2) | 2.2 | (1.5-3.1) | 2.2 | (1.5-3.1) | 2.2 | (1.5-3.1) |
|  | Invasive size | 1.9 | (1.1-2.9) | 1.8 | (1.1-2.8) | 1.8 | (1.1-2.8) | 1.8 | (1.1-2.8) |
| Clinical T | T0 | 25 | ( 0.1%) | 27 | ( 0.1%) | 18 | ( 0.0%) | 20 | ( 0.0%) |
|  | Tis | 2,305 | ( 4.8%) | 2,078 | ( 4.5%) | 2,191 | ( 4.6%) | 2,277 | ( 4.8%) |
|  | T1mi | 2,794 | ( 5.8%) | 2,684 | ( 5.8%) | 2,859 | ( 6.0%) | 2,896 | ( 6.1%) |
|  | T1a | 6,113 | (12.7%) | 5,998 | (13.1%) | 6,332 | (13.3%) | 6,519 | (13.6%) |
|  | T1b | 14,794 | (30.6%) | 14,399 | (31.4%) | 15,033 | (31.5%) | 15,005 | (31.4%) |
|  | T1c | 9,430 | (19.5%) | 8,790 | (19.1%) | 9,044 | (19.0%) | 9,118 | (19.1%) |
|  | T2a | 6,390 | (13.2%) | 5,997 | (13.1%) | 6,182 | (13.0%) | 5,965 | (12.5%) |
|  | T2b | 2,249 | ( 4.7%) | 2,050 | ( 4.5%) | 2,133 | ( 4.5%) | 2,141 | ( 4.5%) |
|  | T3 | 2,889 | ( 6.0%) | 2,622 | ( 5.7%) | 2,629 | ( 5.5%) | 2,638 | ( 5.5%) |
|  | T4 | 1,235 | ( 2.6%) | 1,192 | ( 2.6%) | 1,145 | ( 2.4%) | 1,136 | ( 2.4%) |
|  | TX | 93 | ( 0.2%) | 81 | ( 0.2%) | 94 | ( 0.2%) | 93 | ( 0.2%) |
| Clinical N | N0 | 43,533 | (90.1%) | 41,410 | (90.2%) | 43,192 | (90.6%) | 43,389 | (90.8%) |
|  | N1 | 2,941 | ( 6.1%) | 2,777 | ( 6.0%) | 2,645 | ( 5.5%) | 2,708 | ( 5.7%) |
|  | N2 | 1,527 | ( 3.2%) | 1,464 | ( 3.2%) | 1,535 | ( 3.2%) | 1,434 | ( 3.0%) |
|  | N3 | 106 | ( 0.2%) | 91 | ( 0.2%) | 101 | ( 0.2%) | 101 | ( 0.2%) |
|  | NX | 210 | ( 0.4%) | 176 | ( 0.4%) | 187 | ( 0.4%) | 176 | ( 0.4%) |
| Clinical Stage | 0 | 2,303 | ( 4.8%) | 2,077 | ( 4.5%) | 2,191 | ( 4.6%) | 2,277 | ( 4.8%) |
|  | IA1 | 8,823 | (18.3%) | 8,609 | (18.7%) | 9,116 | (19.1%) | 9,335 | (19.5%) |
|  | IA2 | 14,079 | (29.1%) | 13,718 | (29.9%) | 14,342 | (30.1%) | 14,264 | (29.8%) |
|  | IA3 | 8,484 | (17.6%) | 7,904 | (17.2%) | 8,213 | (17.2%) | 8,249 | (17.3%) |
|  | IB | 5,290 | (10.9%) | 4,937 | (10.8%) | 5,090 | (10.7%) | 4,966 | (10.4%) |
|  | IIA | 1,699 | ( 3.5%) | 1,514 | ( 3.3%) | 1,604 | ( 3.4%) | 1,626 | ( 3.4%) |
|  | IIB | 3,999 | ( 8.3%) | 3,736 | ( 8.1%) | 3,623 | ( 7.6%) | 3,695 | ( 7.7%) |
|  | IIIA | 2,654 | ( 5.5%) | 2,425 | ( 5.3%) | 2,434 | ( 5.1%) | 2,447 | ( 5.1%) |
|  | IIIB | 442 | ( 0.9%) | 434 | ( 0.9%) | 446 | ( 0.9%) | 405 | ( 0.8%) |
|  | IIIC | 12 | ( 0.0%) | 20 | ( 0.0%) | 17 | ( 0.0%) | 17 | ( 0.0%) |
|  | IVA | 348 | ( 0.7%) | 351 | ( 0.8%) | 389 | ( 0.8%) | 348 | ( 0.7%) |
|  | IVB | 76 | ( 0.2%) | 97 | ( 0.2%) | 93 | ( 0.2%) | 78 | ( 0.2%) |
|  | Unconfirmed | 38 | ( 0.1%) | 42 | ( 0.1%) | 37 | ( 0.1%) | 35 | ( 0.1%) |
|  | Occult | 70 | ( 0.1%) | 54 | ( 0.1%) | 65 | ( 0.1%) | 66 | ( 0.1%) |

Table S2. Surgical procedure for lung cancer patients

|  |  | 2019 | | 2020 | | 2021 | | 2022 | |
| --- | --- | --- | --- | --- | --- | --- | --- | --- | --- |
| Approach | RATS | 2,073 | ( 4.3%) | 3,092 | ( 6.7%) | 4,281 | ( 9.0%) | 5,533 | (11.6%) |
|  | VATS | 41,369 | (85.6%) | 37,097 | (80.8%) | 36,838 | (77.3%) | 36,786 | (76.9%) |
|  | Open | 4,875 | (10.1%) | 5,729 | (12.5%) | 6,541 | (13.7%) | 5,489 | (11.5%) |
| Procedure | Pneumonectomy | 283 | ( 0.6%) | 255 | ( 0.6%) | 220 | ( 0.5%) | 177 | ( 0.4%) |
|  | Lobectomy | 33,809 | (70.0%) | 31,061 | (67.6%) | 31,449 | (66.0%) | 30,166 | (63.1%) |
|  | Segmentectomy | 5,501 | (11.4%) | 5,866 | (12.8%) | 6,968 | (14.6%) | 8,177 | (17.1%) |
|  | Wedge | 8,721 | (18.0%) | 8,732 | (19.0%) | 9,016 | (18.9%) | 9,282 | (19.4%) |
| Operation time | (min, IQR) | 175 | (125-231) | 174 | (123-230) | 175 | (124-230) | 173 | (122-227) |
| Blood loss | (ml, IQR) | 21 | (10-80) | 20 | (10-70) | 18 | (10-60) | 13 | (10-50) |
| Length of stay | (days, IQR) | 8 | (6-10) | 7 | (6-10) | 7 | (5-10) | 7 | (5-9) |
| 30-day mortality |  | 171 | ( 0.4%) | 168 | ( 0.4%) | 174 | ( 0.4%) | 163 | ( 0.3%) |
| Complication | Pneumonia | 1,023 | ( 2.1%) | 1,070 | ( 2.3%) | 1,041 | ( 2.2%) | 962 | ( 2.0%) |
|  | Atelectasis | 262 | ( 0.5%) | 251 | ( 0.5%) | 241 | ( 0.5%) | 241 | ( 0.5%) |
|  | Empyema | 389 | ( 0.8%) | 331 | ( 0.7%) | 374 | ( 0.8%) | 326 | ( 0.7%) |
|  | AE of IPF | 270 | ( 0.6%) | 250 | ( 0.5%) | 298 | ( 0.6%) | 220 | ( 0.5%) |
|  | PAL | 2,461 | ( 5.1%) | 2,412 | ( 5.3%) | 2,533 | ( 5.3%) | 2,514 | ( 5.3%) |
|  | Respiratory failure | 202 | ( 0.4%) | 209 | ( 0.5%) | 184 | ( 0.4%) | 154 | ( 0.3%) |

RATS, robot-assisted thoracoscopic surgery; VATS, video-assisted thoracic surgery; IQR, interquartile range; AE, acute exacerbation; IPF, idiopathic pulmonary fibrosis; PAL, prolonged air leak

Table S3. Pathological demographics for lung cancer patients

|  |  | 2019 | | 2020 | | 2021 | | 2022 | |
| --- | --- | --- | --- | --- | --- | --- | --- | --- | --- |
| Tumor size (cm) | Total size | 2.1 | (1.5-3.2) | 2.1 | (1.5-3.1) | 2.1 | (1.5-3.1) | 2.1 | (1.5-3.0) |
|  | Invasive size | 1.7 | (0.9-2.8) | 1.7 | (0.9-2.8) | 1.7 | (0.9-2.8) | 1.7 | (0.9-2.7) |
| Pathology | LUAD | 34,491 | (71.4%) | 32,030 | (69.8%) | 33,542 | (70.4%) | 33,982 | (71.1%) |
|  | LUSQ | 8,669 | (17.9%) | 8,278 | (18.0%) | 8,209 | (17.2%) | 8,133 | (17.0%) |
|  | Others | 5,157 | (10.7%) | 5,610 | (12.2%) | 5,909 | (12.4%) | 5,693 | (11.9%) |
| Pathologica T | Tis | 3,568 | ( 7.4%) | 3,186 | ( 6.9%) | 3,422 | ( 7.2%) | 3,337 | ( 7.0%) |
|  | T0 | 194 | ( 0.4%) | 158 | ( 0.3%) | 179 | ( 0.4%) | 162 | ( 0.3%) |
|  | T1a | 6,117 | (12.7%) | 5,836 | (12.7%) | 6,065 | (12.7%) | 6,129 | (12.8%) |
|  | T1b | 12,059 | (25.0%) | 11,706 | (25.5%) | 12,170 | (25.5%) | 12,314 | (25.8%) |
|  | T1c | 6,541 | (13.5%) | 6,148 | (13.4%) | 6,354 | (13.3%) | 6,444 | (13.5%) |
|  | T1mi | 3,926 | ( 8.1%) | 3,670 | ( 8.0%) | 3,812 | ( 8.0%) | 3,820 | ( 8.0%) |
|  | T2a | 8,997 | (18.6%) | 8,711 | (19.0%) | 9,104 | (19.1%) | 9,095 | (19.0%) |
|  | T2b | 1,969 | ( 4.1%) | 1,840 | ( 4.0%) | 1,931 | ( 4.1%) | 1,928 | ( 4.0%) |
|  | T3 | 3,463 | ( 7.2%) | 3,210 | ( 7.0%) | 3,263 | ( 6.8%) | 3,132 | ( 6.6%) |
|  | T4 | 1,317 | ( 2.7%) | 1,280 | ( 2.8%) | 1,188 | ( 2.5%) | 1,246 | ( 2.6%) |
|  | TX | 109 | ( 0.2%) | 109 | ( 0.2%) | 105 | ( 0.2%) | 126 | ( 0.3%) |
|  | Missing | 57 | ( 0.1%) | 64 | ( 0.1%) | 67 | ( 0.1%) | 75 | ( 0.2%) |
| Pathological N | N0 | 39,101 | (80.9%) | 37,013 | (80.6%) | 38,957 | (81.7%) | 39,197 | (82.0%) |
|  | N1 | 3,362 | ( 7.0%) | 3,270 | ( 7.1%) | 3,161 | ( 6.6%) | 3,122 | ( 6.5%) |
|  | N2 | 3,363 | ( 7.0%) | 3,210 | ( 7.0%) | 3,118 | ( 6.5%) | 3,119 | ( 6.5%) |
|  | N3 | 71 | ( 0.1%) | 61 | ( 0.1%) | 60 | ( 0.1%) | 67 | ( 0.1%) |
|  | NX | 2,363 | ( 4.9%) | 2,300 | ( 5.0%) | 2,296 | ( 4.8%) | 2,230 | ( 4.7%) |
|  | Missing | 57 | ( 0.1%) | 64 | ( 0.1%) | 68 | ( 0.1%) | 73 | ( 0.2%) |
| Pathological Stage | 0 | 3,565 | ( 7.4%) | 3,184 | ( 6.9%) | 3,418 | ( 7.2%) | 3,334 | ( 7.0%) |
|  | IA1 | 9,840 | (20.4%) | 9,340 | (20.3%) | 9,692 | (20.3%) | 9,768 | (20.4%) |
|  | IA2 | 10,963 | (22.7%) | 10,697 | (23.3%) | 11,129 | (23.4%) | 11,354 | (23.7%) |
|  | IA3 | 5,404 | (11.2%) | 5,046 | (11.0%) | 5,389 | (11.3%) | 5,441 | (11.4%) |
|  | IB | 6,511 | (13.5%) | 6,354 | (13.8%) | 6,690 | (14.0%) | 6,687 | (14.0%) |
|  | IIA | 1,355 | ( 2.8%) | 1,214 | ( 2.6%) | 1,353 | ( 2.8%) | 1,335 | ( 2.8%) |
|  | IIB | 4,720 | ( 9.8%) | 4,496 | ( 9.8%) | 4,430 | ( 9.3%) | 4,309 | ( 9.0%) |
|  | IIIA | 3,952 | ( 8.2%) | 3,586 | ( 7.8%) | 3,559 | ( 7.5%) | 3,633 | ( 7.6%) |
|  | IIIB | 744 | ( 1.5%) | 780 | ( 1.7%) | 735 | ( 1.5%) | 718 | ( 1.5%) |
|  | IIIC | 9 | ( 0.0%) | 11 | ( 0.0%) | 10 | ( 0.0%) | 14 | ( 0.0%) |
|  | IVA | 809 | ( 1.7%) | 801 | ( 1.7%) | 832 | ( 1.7%) | 797 | ( 1.7%) |
|  | IVB | 90 | ( 0.2%) | 94 | ( 0.2%) | 88 | ( 0.2%) | 76 | ( 0.2%) |
|  | Unconfirmed | 223 | ( 0.5%) | 184 | ( 0.4%) | 209 | ( 0.4%) | 189 | ( 0.4%) |
|  | Occult | 74 | ( 0.2%) | 65 | ( 0.1%) | 58 | ( 0.1%) | 78 | ( 0.2%) |
|  |  | 58 | ( 0.1%) | 66 | ( 0.1%) | 68 | ( 0.1%) | 75 | ( 0.2%) |

LUAD, lung adenocarcinoma, LUSQ, lung squamous cell carcinoma

Table S4. Characteristics of mediastinal tumor patients

|  |  | 2019 | | 2020 | | 2021 | | 2022 | |
| --- | --- | --- | --- | --- | --- | --- | --- | --- | --- |
| Age | -59 | 1,526 | (39.0%) | 1,462 | (39.2%) | 1,493 | (40.0%) | 1,489 | (38.4%) |
|  | 60-69 | 1,014 | (25.9%) | 890 | (23.9%) | 917 | (24.6%) | 960 | (24.8%) |
|  | 70-79 | 1,107 | (28.3%) | 1,115 | (29.9%) | 1,045 | (28.0%) | 1,135 | (29.3%) |
|  | 80- | 264 | ( 6.8%) | 264 | ( 7.1%) | 280 | ( 7.5%) | 293 | ( 7.6%) |
| Gender | Female | 1,987 | (50.8%) | 1,897 | (50.8%) | 1,890 | (50.6%) | 1,933 | (49.9%) |
|  | Male | 1,924 | (49.2%) | 1,834 | (49.2%) | 1,845 | (49.4%) | 1,944 | (50.1%) |
| Performance status | PS0 | 3,476 | (88.9%) | 3,295 | (88.3%) | 3,345 | (89.6%) | 3,495 | (90.1%) |
|  | PS1 | 316 | ( 8.1%) | 324 | ( 8.7%) | 292 | ( 7.8%) | 281 | ( 7.2%) |
|  | PS2-4 | 119 | ( 3.0%) | 112 | ( 3.0%) | 98 | ( 2.6%) | 101 | ( 2.6%) |
| MG |  | 344 | ( 8.8%) | 359 | ( 9.6%) | 374 | (10.0%) | 377 | ( 9.7%) |
|  |  |  |  |  |  |  |  |  |  |
| Approach | RATS | 507 | (13.0%) | 629 | (16.9%) | 858 | (23.0%) | 1,076 | (27.8%) |
|  | VATS | 1,841 | (47.1%) | 1,677 | (44.9%) | 1,633 | (43.7%) | 1,593 | (41.1%) |
|  | Sternotomy | 1,196 | (30.6%) | 1,077 | (28.9%) | 967 | (25.9%) | 933 | (24.1%) |
|  | Others | 296 | ( 0.8%) | 296 | ( 0.8%) | 232 | ( 0.6%) | 213 | (0.6%) |
| Procedure | Thymectomy | 993 | (25.4%) | 926 | (24.8%) | 1,054 | (28.2%) | 1,008 | (26.0%) |
|  | Extended thymectomy | 765 | (19.6%) | 721 | (19.3%) | 661 | (17.7%) | 675 | (17.4%) |
|  | Subtotal thymectomy | 81 | ( 2.1%) | 79 | ( 2.1%) | 102 | ( 2.7%) | 83 | ( 2.1%) |
|  | Tumorectomy | 2,072 | (53.0%) | 2,005 | (53.7%) | 1,918 | (51.4%) | 2,111 | (54.4%) |
|  |  |  |  |  |  |  |  |  |  |
| Operation time | (min, IQR) | 139 | (93-203) | 140 | (93-204) | 145 | (97-211) | 143 | (93-205) |
| Blood loss | (ml, IQR) | 10 | (10-100) | 10 | (10-100) | 10 | (10-78) | 10 | (10-75) |
|  |  |  |  |  |  |  |  |  |  |
| Length of stay | (days, IQR) | 6 | (4-8) | 6 | (4-8) | 6 | (4-8) | 5 | (4-8) |
| 30-day mortality |  | 2 | ( 0.1%) | 2 | ( 0.1%) | 7 | ( 0.2%) | 7 | ( 0.2%) |
| Complication | Pneumonia | 23 | ( 0.6%) | 23 | ( 0.6%) | 24 | ( 0.6%) | 25 | ( 0.6%) |
|  | Atelectasis | 13 | ( 0.3%) | 11 | ( 0.3%) | 10 | ( 0.3%) | 6 | ( 0.2%) |
|  | Empyema | 7 | ( 0.2%) | 5 | ( 0.1%) | 8 | ( 0.2%) | 3 | ( 0.1%) |
|  | Respiratory failure | 30 | ( 0.8%) | 34 | ( 0.9%) | 28 | ( 0.7%) | 16 | ( 0.4%) |

MG, myasthenia gravis; RATS, robot-assisted thoracoscopic surgery; VATS, video-assisted thoracic surgery; IQR, interquartile range

Table S5. Pathological demographics of mediastinal tumor patients

|  |  | 2019 | | 2020 | | 2021 | | 2022 | |
| --- | --- | --- | --- | --- | --- | --- | --- | --- | --- |
| Tumor size | (cm, IQR) | 4 | (2.5-6) | 4 | (2.5-6) | 4 | (2.5-6) | 4 | (2.3-6) |
| Histology | Thymoma | 2,322 | (59.4%) | 2,290 | (61.4%) | 2,253 | (60.3%) | 2,325 | (60.0%) |
|  | TC | 357 | ( 9.1%) | 339 | ( 9.1%) | 392 | (10.5%) | 387 | (10.0%) |
|  | TNET | 45 | ( 1.2%) | 48 | ( 1.3%) | 48 | ( 1.3%) | 57 | ( 1.5%) |
|  | GCT(Malignant) | 21 | ( 0.5%) | 18 | ( 0.5%) | 25 | ( 0.7%) | 28 | ( 0.7%) |
|  | Malignant lymphoma | 30 | ( 0.8%) | 17 | ( 0.5%) | 13 | ( 0.3%) | 22 | ( 0.6%) |
|  | GCT(Benign) | 85 | ( 2.2%) | 68 | ( 1.8%) | 81 | ( 2.2%) | 53 | ( 1.4%) |
|  | Cyst | 911 | (23.3%) | 808 | (21.7%) | 769 | (20.6%) | 820 | (21.2%) |
|  | Others | 140 | ( 0.4%) | 143 | ( 0.4%) | 154 | ( 0.4%) | 185 | ( 0.5%) |

IQR, interquartile range; TC, thymic carcinoma, TNET, Neuroendocrine tumors of the thymus; GCT, germ cell tumor
